# Metformin preferentially enhances the radio-sensitivity of cancer stem-like cells with highly mitochondrial respiration ability in HMPOS

**DOI:** 10.1016/j.omto.2021.08.007

**Published:** 2021-08-19

**Authors:** Tatsuya Deguchi, Kenji Hosoya, Shango Kim, Yusuke Murase, Kumiko Yamamoto, Tomoki Bo, Hironobu Yasui, Osamu Inanami, Mahiro Okumura

**Affiliations:** 1 Laboratory of Veterinary Surgery, Department of Clinical Sciences, Graduate School of Veterinary Medicine, Hokkaido University, N18 W9 Sapporo, Hokkaido 060-0818, Japan; 2 Laboratory of Radiation Biology, Department of Applied Veterinary Science, Graduate School of Veterinary Medicine, Hokkaido University, N18 W9 Sapporo, Hokkaido 060-0818, Japan

**Keywords:** metformin, radiation, cancer stem-like cells, dog, mitochondria

## Abstract

Metformin has many anti-cancer effects, alone or in combination with radiation. However, the mechanism underlying its radio-sensitized effect is still unclear, especially for cancer stem-like cells (CSCs). Here, the radio-sensitized effect of metformin was investigated, and its mechanism was revealed in CSCs derived from canine osteosarcoma cell line (HMPOS), a canine osteosarcoma cell line. Spheroid cells (SCs) were used as CSCs-rich cells derived from sphere formation, and SCs were compared with normal adherent culture cells (ACs). The radio-sensitizing effect of metformin using clonogenic assay and tumor growth in mice xenograft model were evaluated, and the mechanism of its radio-sensitization focusing on mitochondrial function was revealed. Metformin significantly enhanced radio-sensitivity of SCs through its inhibition of the mitochondrial function, as shown by decreased oxygen consumption, decreased mitochondrial membrane potential, and decreased ATP production. Additionally, SCs had a higher ability of mitochondrial respiration than ACs, which may have caused difference of their sensitivity of metformin and irradiation. In conclusion, mitochondrial function might play an important role in the sensitivity of metformin and irradiation, and drugs that target mitochondrial respiration, such as metformin, are promising radio-sensitizers to target CSCs.

## Graphical abstract

**Figure:**
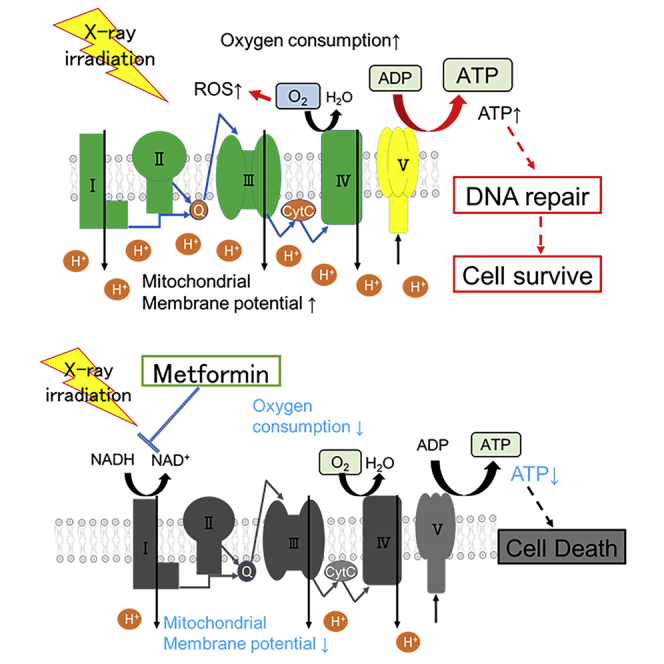

## Teaser

Metformin preferentially radio-sensitized spheroid cells (SCs) of canine osteosarcoma cell line, which has cancer stem-like cells (CSCs) properties. SCs had a higher ability of mitochondrial function, which might account for differences in sensitivity of metformin and irradiation. Drugs targeting mitochondrial respiration, such as metformin, are promising radio-sensitizers in CSCs.

## Introduction

Solid tumors are composed of heterogeneous cancer cells and contain a small subpopulation of cancer stem-like cells (CSCs).^1^ It has been demonstrated CSCs are characterized to be self-renewing cells in tumors that generate differentiated progeny by the differentiation to CSCs and non-CSCs and unlimited proliferative capacity.^2^ Several studies have indicated that CSCs are more resistant to radiation therapy than non-CSCs, and their survival after radiation therapy has been linked to cancer recurrence.^3^

Induction of sphere formation is considered a valuable method to maintain cell viability during isolation of CSCs from cancer tissues and cell lines.^4^^,^^5^ Principally, sphere formation occurs through ultra-low attachment condition, which contributes to limiting normal cell growth, proliferation, and differentiation. In addition, epidermal growth factors (EGFs) and fibroblast growth factors (FGFs) are cooperated to maintain the stemness characteristics and create tumor spheres.^6^^,^^7^ We previously established spheroid cells (SCs) derived from canine cancer cell lines using sphere formation, which had CSC-like properties, including high expression level of CD133, high tumorigenesis capacity, and radio-resistance compared with adherent cells (ACs), which cultured normal adherent condition.^8^

Recently, metformin has gained attention as one of the promising anti-cancer drugs that can enhance tumor cell’s radio-sensitivity.^9^^,^^10^ Metformin targets the mitochondrial respiratory complex 1, which leads to membrane depolarization, release of reactive oxygen species (ROS), decrease in oxygen consumption, decrease in mitochondrial membrane potential, and decrease in the ATP/ADP ratio.^11^^,^^12^ Inhibition of mitochondrial complex and changing energy depletion activates 5′ adenosine-monophosphate-activated protein kinases (AMPKs), which suppress the mammalian target of rapamycin (mTOR) pathway.^13^ However, results of radio-enhancement mechanism by metformin are controversial, especially in case of CSCs. For example, Lonardo et al.^14^ showed that metformin’s effect mostly relied on inhibition of mitochondrial function, which apparently was lethal for CSCs both *in vitro* and *in vivo*. However, Song et al.^10^ showed that metformin and ionizing radiation activated AMPKs, leading to inactivation of mTOR and suppression of its downstream effectors on CSCs. The mechanism of metformin thought which radio-sensitize CSCs remains an area of active investigation. Moreover, metformin was more effective against cells having a high mitochondrial respiration level. However, the factors determining radio-sensitizing effect of metformin have not been elucidated to date.

This study determined the radio-sensitization effects of metformin both *in vitro* and *in vivo*, including investigation of its radio-sensitization mechanism and difference of radio-sensitizing efficiencies between SCs and their parental ACs. Metformin preferentially radio-sensitized SCs, leading to inhibition of mitochondrial respiration, but not to AMPK activation. Additionally, SCs had a higher ability of mitochondrial respiration than ACs, which might cause the difference of radio-sensitization effect of metformin. In summary, mitochondrial respiration might play a central role in the radio-resistance mechanism of CSCs and metformin is a promising radio-sensitizer that can inhibit mitochondrial respiration of CSCs.

## Results

### Metformin inhibited sphere formation and sensitized SCs to radiation *in vitro*

**Figure 1:**
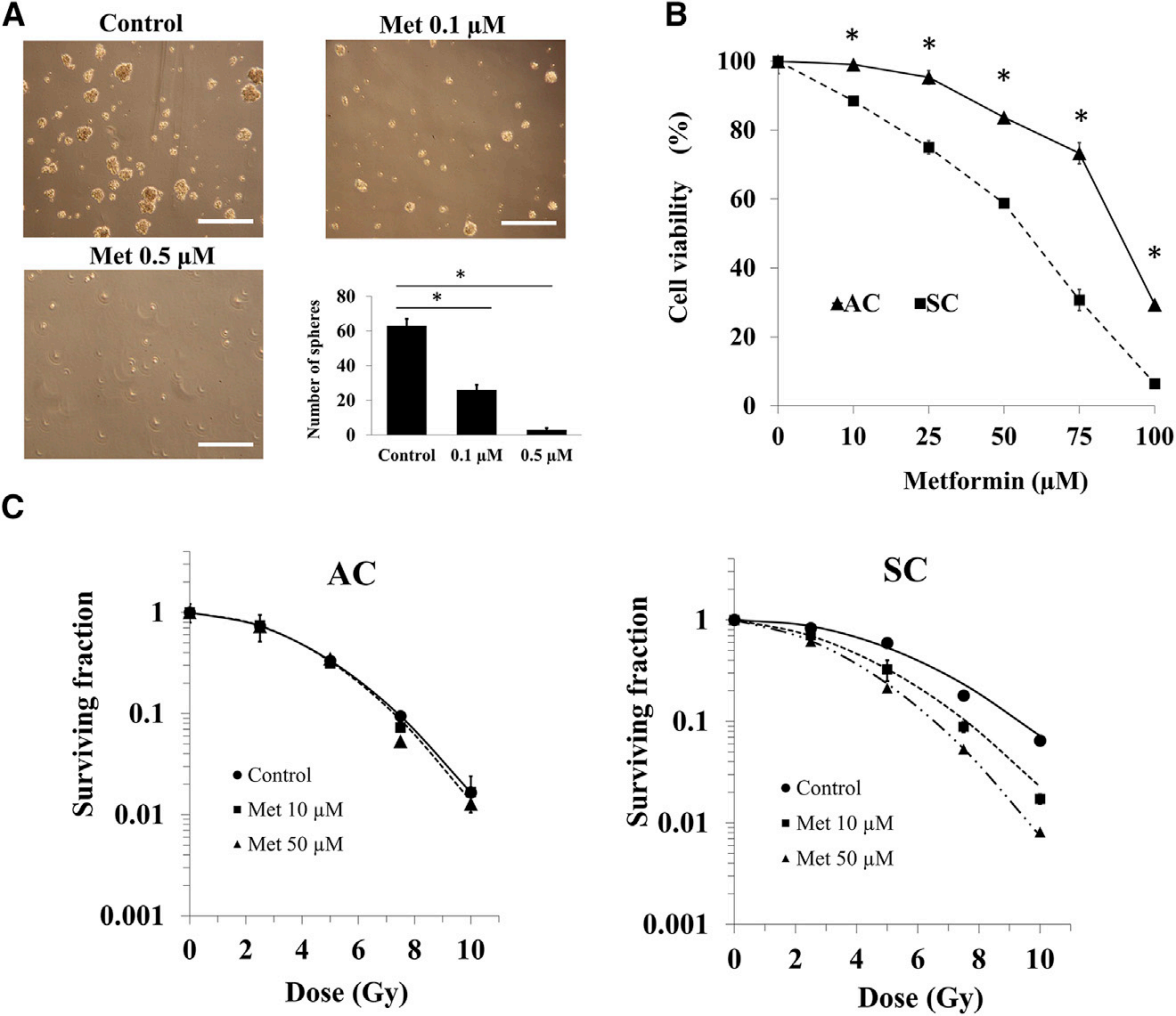
Inhibition of sphere formation by metformin and survival curves of ACs and SCs treated X-irradiation with or without metformin (A) Effects of metformin for sphere formation. Scale bar: 500 μm. (B) Cell viability of treatment with 10–100 μM metformin for 24 h in ACs and SCs is shown. (C) Survival curves of X-irradiation in ACs and SCs treated with or without metformin are shown. These results were analyzed using Mann-Whitney *U* test. ∗p < 0.05 for with versus without metformin.

After 10 days of incubation without metformin, 63 ± 4 spheres were formed, although 26 ± 3 and 3 ± 1 were formed when incubated with 0.1 and 0.5 μM metformin, respectively (Figure 1A). Exposure to metformin for 24 h reduced the clonogenic survival of ACs and SCs in a dose-dependent manner. The sensitivity of SCs to metformin was significantly higher compared to that of ACs (Figure 1B). After X-irradiation with 10 μM and 50 μM metformin, the colony formation of ACs and SCs was measured. The survival curve of SCs treated with metformin and X-irradiation was steeper than that of SCs treated with X-irradiation alone. In SCs, metformin showed significantly enhanced cell death induced by X-irradiation compared to control cells without metformin, whereas in ACs, there was no significant effect of metformin (Figure 1C).

### Metformin, X-ray irradiation, and their combination induced intracellular ROS production

**Figure 2:**
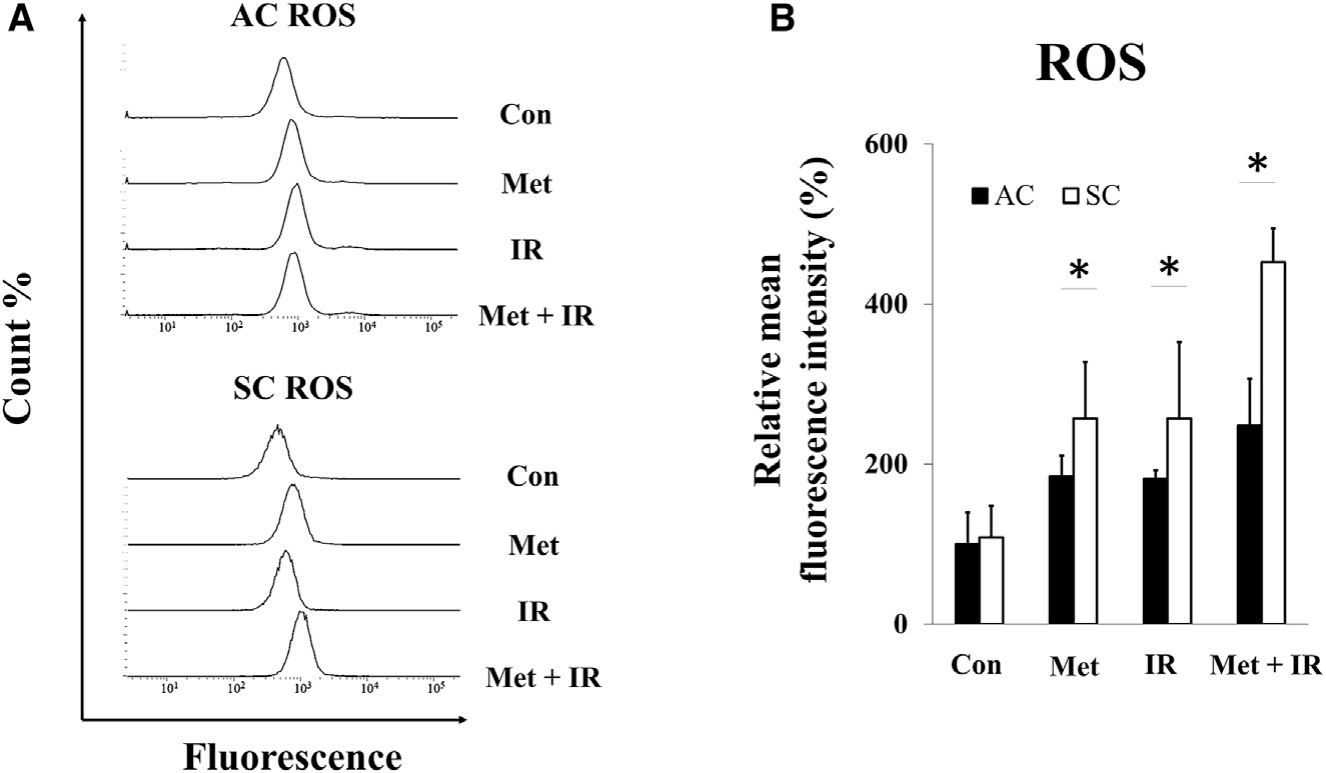
Intracellular ROS in ACs and SCs (A) Histogram of ACs and SCs of control (Con), 24 h exposure with 50 μM metformin (Met), 5 Gy of X-irradiation (IR), and both metformin plus X-irradiation (Met + IR). (B) Relative mean fluorescence intensity (%) in ACs and SCs is shown. These results were analyzed using Mann-Whitney *U* test. ∗p < 0.05 for ACs versus SCs.

Changes in the levels of intracellular ROS by the treatment of metformin, X-irradiation, and metformin plus X-irradiation were evaluated. In ACs and SCs treated with metformin, X-irradiation, and metformin plus X-irradiation, increased ROS determined by the increase in the fluorescence intensity, compared to that in control, and the intensity in treatment with metformin plus X-irradiation of SC was far higher than metformin or X-irradiation alone (Figure 2A). The difference in relative mean fluorescence intensity (MFI) between treatment (metformin, X-irradiation, and metformin plus X-irradiation) and control groups was significantly higher in both ACs and SCs. However, ROS levels of SCs treated with metformin, X-irradiation, and metformin plus X-irradiation were significantly higher than those of ACs (Figure 2B).

### Metformin inhibited mitochondrial respiration, activated by X-ray irradiation

**Figure 3:**
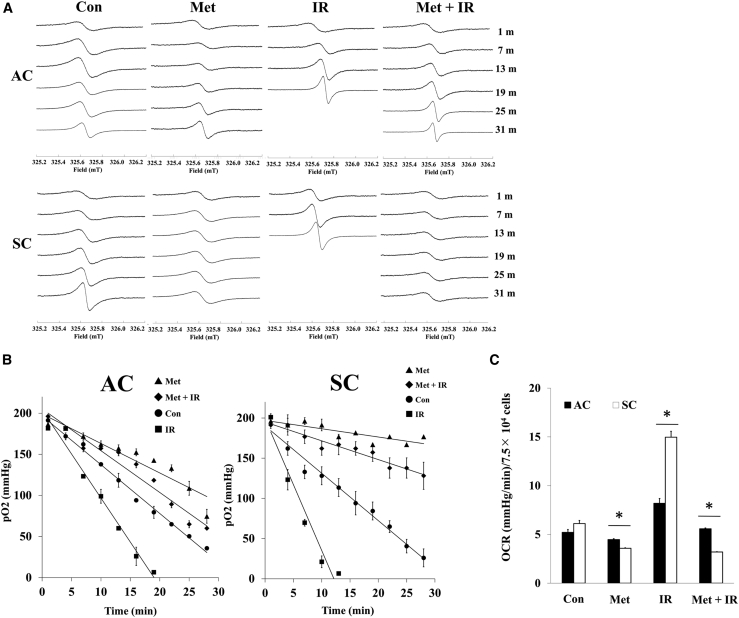
ESR analysis of oxygen consumption in ACs and SCs (A and B) Representative ESR spectra (A) changes in pO_2_ (B) of ACs and SCs treated with 50 μM Met, 5 Gy of IR, and Met + IR. (C) OCR in ACs and SCs is shown. These results were analyzed using Mann-Whitney *U* test. ∗p < 0.05 for ACs versus SCs.

The intracellular ROS are considered important parameters, but activation and inhibition of mitochondrial respiration cause increment of ROS production. Therefore, oxygen consumption was investigated to evaluate mitochondrial respiration. The oxygen consumption ratio (OCR) of ACs and SCs without any treatment (control) was calculated to be 5.21 ± 0.30 and 6.12 ± 0.31 mmHg/min/7.5 × 10^4^ cells, respectively, by the linear relationship between pO_2_ and time (Figures 3A and 3B). Metformin inhibited cellular oxygen consumption in ACs and SCs compared to control, 4.46 ± 0.09 and 3.58 ± 0.05 mmHg/min/7.5 × 10^4^ cells, respectively. X-irradiation, on the other hand, increased cellular oxygen consumption in ACs and SCs compared to control, 8.19 ± 0.49 and 14.67 ± 0.61 mmHg/min/7.5 × 10^4^ cells, respectively. These changes in SCs were significantly higher than those in ACs. Additionally, X-irradiation-induced oxygen consumption was decreased in SCs treated with metformin plus X-irradiation, and OCR of SCs (3.19 ± 0.03) treated with metformin plus X-irradiation was significantly lower than that of control. However, OCR of ACs treated with metformin plus X-irradiation (5.58 ± 0.08) was comparable with control (Figure 3C). According to these results, in SCs, metformin inhibits irradiation-induced activation of mitochondrial respiration and decreases activated oxygen consumption as lower than control.

### Metformin inhibited ATP production induced by X-ray irradiation in SCs

**Figure 4:**
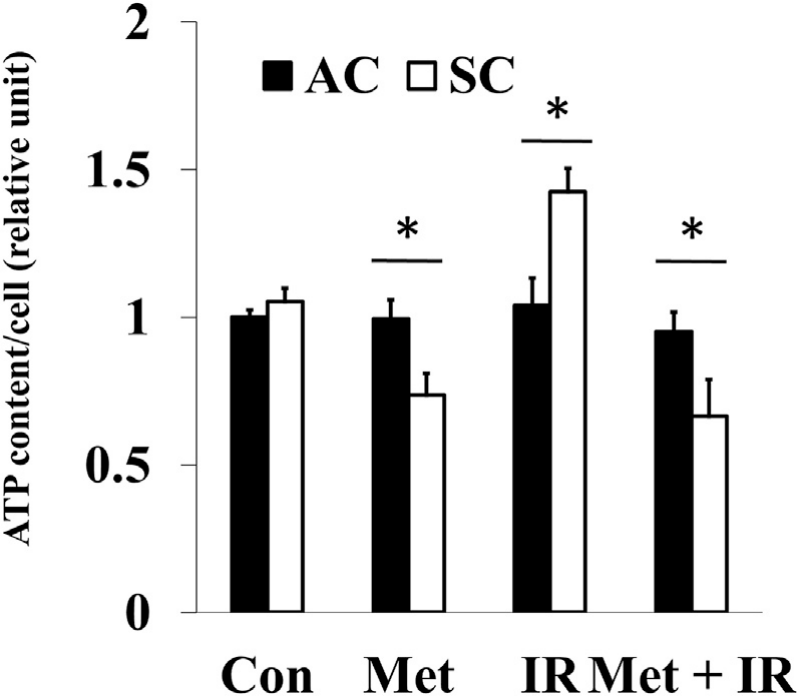
Cellular ATP content in ACs and SCs ATP contents of ACs and SCs treated with histogram of ACs and SCs with 50 μM Met, 5 Gy of IR, and Met + IR. These results were analyzed using Mann-Whitney *U* test. ∗p < 0.05 for ACs versus SCs.

Mitochondrial respiration is associated with cellular energy production. Metformin decreased ATP production, and X-irradiation increased ATP production in SCs. Moreover, ATP production of metformin plus X-irradiation decreased significantly more than that of control. However, in ACs, there were no significant differences in ATP production between control and treatment (metformin, X-irradiation, and metformin plus X-irradiation; Figure 4). These data suggested that metformin and X-irradiation affect mitochondrial energy production in SCs.

### X-irradiation increased mitochondrial membrane potential in SCs

**Figure 5:**
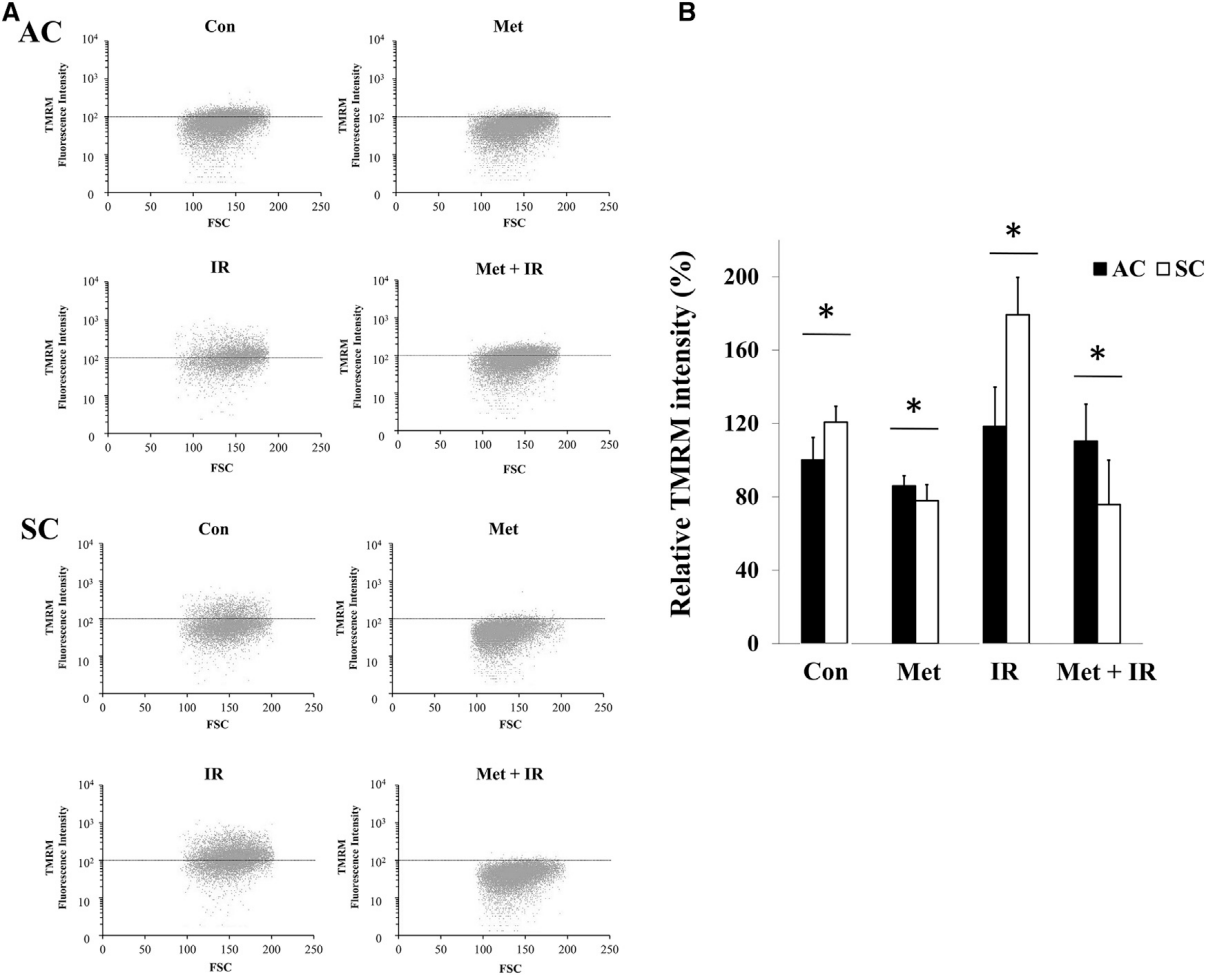
Mitochondrial membrane potential in ACs and SCs (A and B) Forward scatter (FSC) and fluorescein isothiocyanate (FITC) dot plot analysis of ACs (A) and SCs (B) of Con, 24 h exposure with 50 μM Met, 5 Gy of IR, and Met + IR. The dot horizontal line represents 10^2^ TMRM fluorescence intensity. (C) Relative TMRM intensity (%) in ACs and SCs is shown. These results were analyzed using Mann-Whitney *U* test. ∗p < 0.05 for ACs versus SCs.

The mitochondrial membrane potential, which indicates mitochondrial electron transport chain (ETC) activity, was investigated. X-irradiation increased tetramethyl rhodamine methyl ester (TMRM) fluorescence intensity compared to respective control in ACs and SCs, whereas metformin decreased the intensity except for that in ACs with metformin plus X-irradiation (Figures 5A and 5B). The difference in relative MFI between ACs and SCs was statistically significant in control, X-irradiation, and metformin plus X-irradiation (Figure 5C). These results suggested that SCs had higher mitochondrial membrane potential under normal and irradiation conditions than ACs, and metformin decreased mitochondrial membrane potential, especially in irradiated SCs.

### SCs had a higher mitochondrial respiration capacity

**Figure 6:**
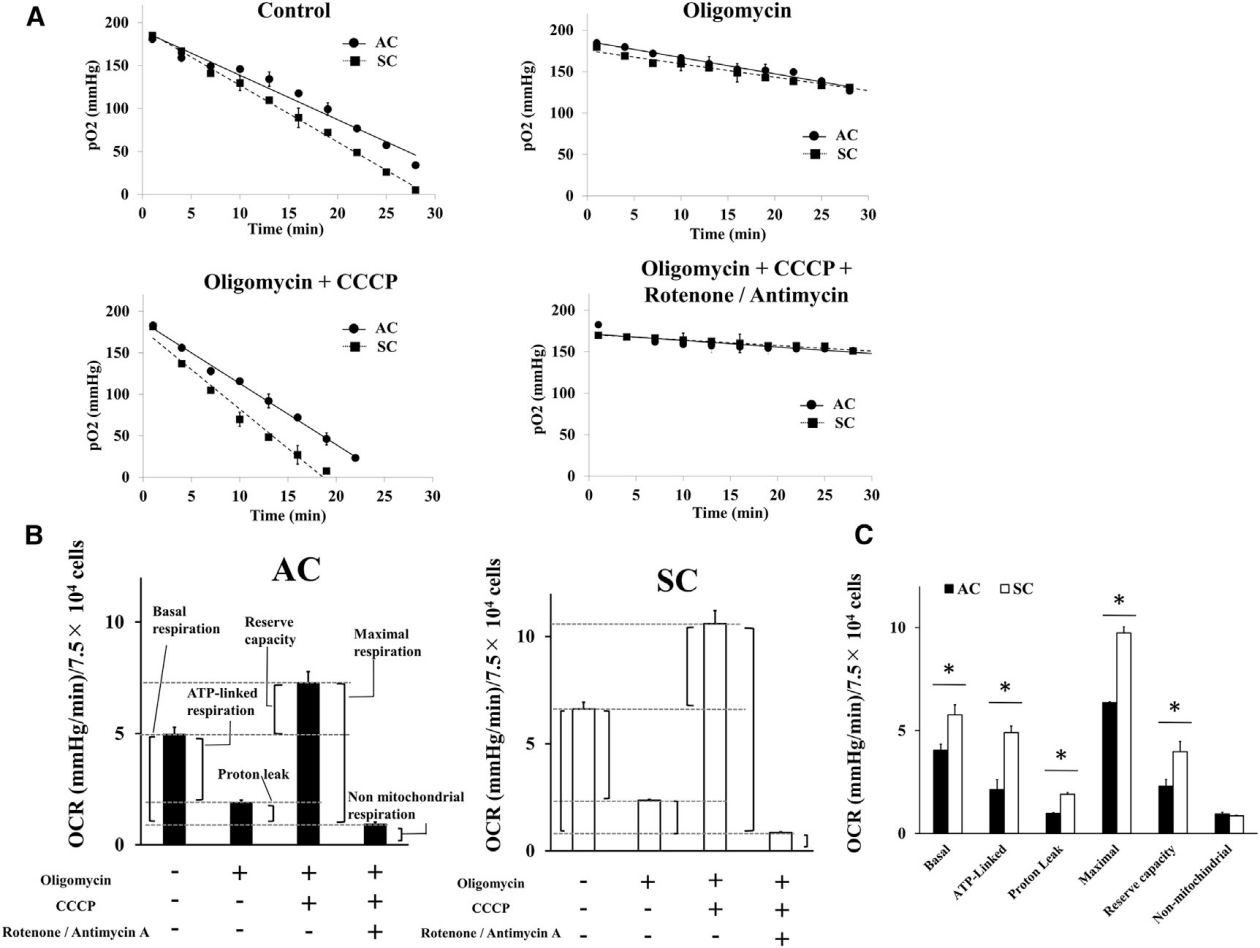
Mitochondrial respiratory function of ACs and SCs (A) Changes in pO_2_ of ACs and SCs in the absence and the presence of mitochondria-targeting reagent. (B and C) OCR in ACs and SCs in the absence and presence of a variety of mitochondria-targeting reagents is shown. These results were analyzed using Mann-Whitney *U* test. ∗p < 0.05 for ACs versus SCs.

To elucidate the difference in sensitivity to metformin and irradiation between ACs and SCs, the respiratory parameters were calculated as previously described using electron spin resonance (ESR), oximetry by adding mitochondria-targeting reagents. The following four patterns of metabolic inhibitors were evaluated: (1) without any reagents; (2) oligomycin A (1 μM); (3) oligomycin + carbonyl cyanide m-chlorophenyl hydrazine (CCCP) (1 μM); and (4) oligomycin + CCCP + combination of rotenone (1 μM) and antimycin A (1 μM; Figure 6A). The respiratory parameters, including basal respiration, ATP-linked respiration, proton leak, maximal respiration, reserve capacity, and non-mitochondrial respiration, were calculated and summarized (Figure 6B). Total mitochondrial ETC function of SCs, including basal respiration, ATP-linked respiration, proton leak, maximal respiration, and reserve capacity, was significantly higher than that of ACs (Figure 6C).

### Combination of metformin and radiation enhanced suppression of tumor growth

**Figure 7:**
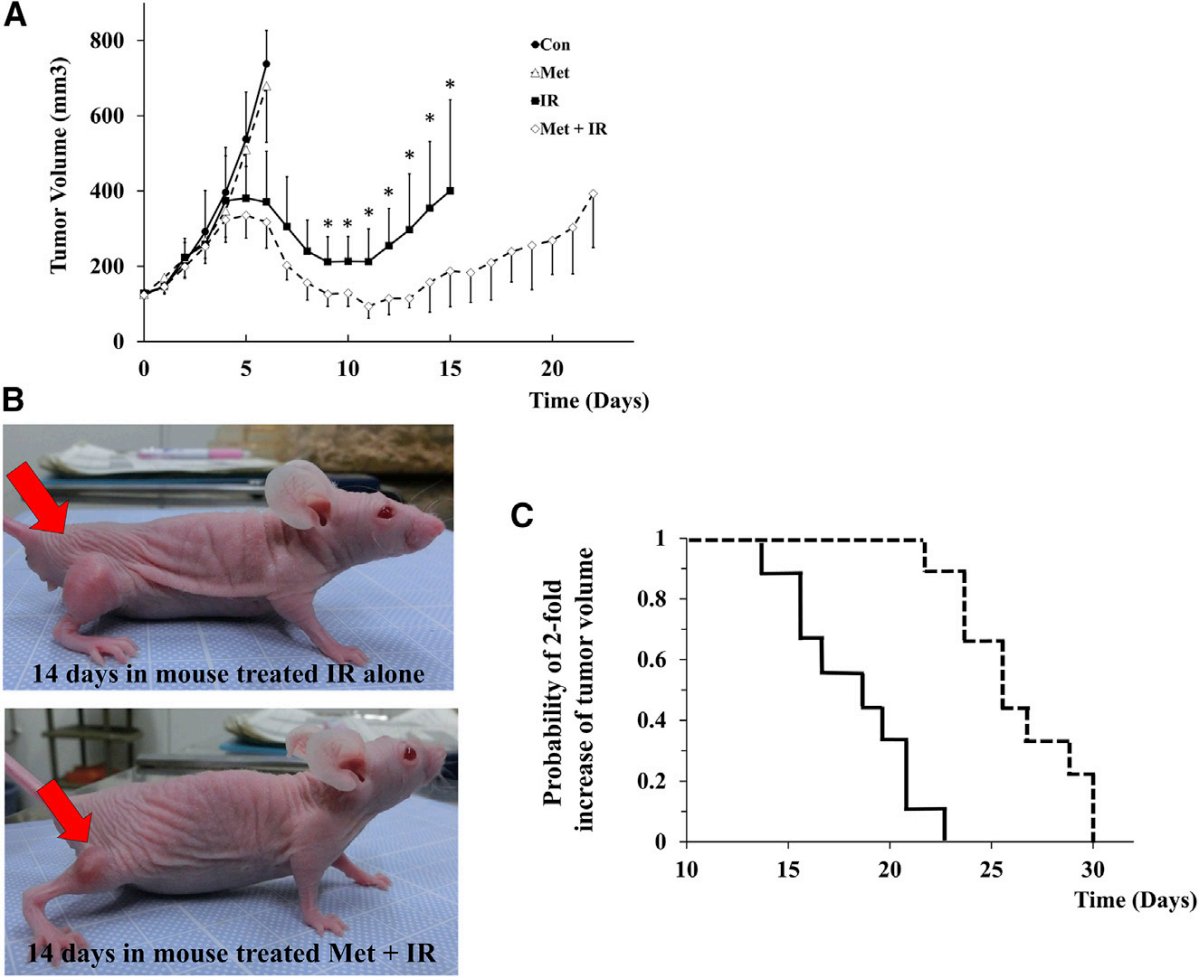
Tumor growth inhibition in xenograft mice (A and B) Tumors grown to 100 mm^3^ of mice were treated as follows: Con, Met (25 mg/kg twice a day injected by i.p.), 20 Gy of IR, and Met + IR. The Kaplan-Meier curves for 2-fold increase of tumor volume after assigning are shown. In IR and Met + IR, the median time for 2-fold increase of tumor volume was 18 and 25 days, respectively. Results of tumor volume were analyzed using Mann-Whitney *U* test. ∗p < 0.05 for IR versus Met + IR.

Tumors of mice with metformin treatment (25 mg/kg twice a day) did not reduce the tumor growth compared to control (no treatment). On the other hand, 20-Gy irradiation markedly decreased tumor volume. The tumor volume of mice treated with metformin plus X-irradiation was significantly smaller than that of mice treated with X-irradiation alone (Figures 7A and 7B). Moreover, the days required for 2-fold increase of tumor volume in mice treated with X-irradiation alone and metformin plus X-irradiation were 18 (13–22) and 25 (21–29) days after assigning (Figure 7C). The difference of the days required for 2-fold increase was statistically longer in mice treated with metformin plus X-irradiation than in that with X-irradiation alone.

## Discussion

Metformin is known to inhibit mitochondrial complex in the mitochondrial ETC chain, yet it is currently unknown whether the anti-cancer effect of metformin occurs through inhibition of the mitochondrial complex. The present study evidence that metformin enhanced decrease of clonogenic cell survival of SCs after irradiation (Figure 1) through inhibition of mitochondrial respiration (Figure 3). Additionally, this effect of metformin was independent of AMPK phosphorylation (Figure S2). It was demonstrated that there were differences in mitochondrial respiration capacity between SCs and ACs (Figure 6). These differences in mitochondrial function might be linked to sensitivity to anti-cancer effect of metformin and irradiation. Furthermore, clinically achievable dose of metformin treatment delayed tumor regrowth after irradiation in mice transplanted with SCs (Figure 7). For the clinical application, the dose used for mice in this study was effective *in vivo* and suggested to be a recommended safe dose in human and animals.

Metformin inhibited sphere formation, and clonogenic survival of SCs was significantly lower compared to that of ACs in each dose of metformin (Figures 1A and 1B). It has been suggested that metformin and this analog inhibits sphere formation, and CSCs have been more sensitive to metformin than non-CSCs in pancreatic cancer cells.^15^ The difference of sensitivity to metformin is associated with the AMPK/mTOR pathways.^16^ However, there was no difference expression level of AMPK/mTOR between SCs and ACs. Further analysis that evaluated metformin uptake or organic caution transporters levels in CSCs is needed.

Exposure to metformin for 24 h at 50 μM concentration was preferentially cytotoxic and radio-sensitized SCs through inhibiting mitochondrial respiration and independently on AMPK activation (Figure S2). It has been reported that metformin killed CSCs through the activation of AMPK, which affects its downstream pathway (mainly mTOR signaling pathway), but these studies used 1–50 mM metformin and exposure time was over 24 h.^10^^,^^13^^,^^17^ Exposure to lower concentrations of metformin, 5–25 μM, for 1–72 h enhanced radiation-induced cell death, whereas AMPK activation reached high levels after 48–72 h of metformin exposure.^18^ According to these results, AMPK activation by metformin is a secondary phenomenon that occurred after the decrease of ATP production by mitochondrial respiration, and low concentration and short exposure time of metformin can radio-sensitize cancer cells through inhibiting the mitochondrial respiration, but not AMPK activation.^11^^,^^12^

It was demonstrated that metformin induces a lethal energy crisis by enhancing ROS production and reducing ATP levels and mitochondrial membrane potential in SCs (Figures 2, 4, and 5). Furthermore, it was also found that metformin inhibits cellular oxygen consumption as an indicator of metformin toxicity at the cellular level in SCs (Figure 3). Increments of intracellular ROS levels are involved in DNA damage, disturbing cell redox balance, and signal transduction pathways.^18^ It has been suggested that metformin increases intracellular ROS level, thus providing an alternative mechanism to AMPK activation.^19^ Metformin also decreased mitochondrial respiration, membrane potential, and ATP production, suggesting it causes suppression of ETC function. Recent research has demonstrated a role for the inhibition of mitochondrial complex in the anti-cancer effect of metformin.^20^^,^^21^ The improving mitochondrial-targeting metformin analogs are much more potent than metformin in inhibiting cancer cells.^16^ These results suggested that the anti-cancer effect of metformin depends on the inhibition of the mitochondrial complex and disturbing metabolism of mitochondria.

To elucidate the difference in metformin sensitivity between ACs and SCs, the mitochondrial function was compared using ESR oximetry. In this study, SCs had higher mitochondrial function than ACs under both non-irradiation and irradiation condition, which was indicated by high ability of mitochondrial basal, ATP-linked, maximal respiration, and proton leak in SCs. This result suggested SCs had a highly level of oxidative phosphorylation (Figure 6). It is known that cancer cells preferentially use anaerobic glycolysis compared to normal tissue, which is referred to as Warburg effect, but metabolic features of CSCs have still remained controversial.^22^ Several reports have shown that CSCs rely more on oxidative phosphorylation than on glycolysis for their energy supply.^23^, ^24^, ^25^ This observation can explain why metformin is more effective for CSC because metformin is more effective in cells with high cellular oxidative phosphorylation, which correlated with high ability of mitochondrial respiration.

To our knowledge, it has reported for the first time that SCs have high ability for mitochondrial respiration and can activate their respiration when irradiated, which induces intracellular ROS and ATP production, and increase mitochondrial membrane potential (Figures 2, 3, 4, and 5). Irradiation increases oxidative phosphorylation of cells, which have high mitochondrial reserve capacity.^26^ Reserve capacity indicates ability of cells to increase oxidative phosphorylation in order to respond to stress, such as irradiation.^27^ It is still unknown whether the activation of mitochondrial metabolism contributes to cell-fate decisions, but several reports demonstrated that mitochondrial ATP production enhanced cell survival after irradiation.^28^ Furthermore, SCs had a high DNA repair capacity and metformin inhibited DNA repair in SCs (Figure S1). These results showed that mitochondrial function and ATP production were linked with radio-resistance of cancer cells. For example, ATP-dependent chromatin remodeling complexes in double-strand break (DSB) repair and poly ADP-ribose polymerase (PARP) activity are ATP dependent.^29^, ^30^, ^31^, ^32^, ^33^ Thus, mitochondrial function might play an important role in cell’s radio-sensitivity, and high mitochondrial respiratory capacity of CSCs might be linked with their radio-resistance through DNA repair.

Metformin dosage for anti-cancer effect was not clarified in humans and dogs. However, a dose of 10–15 mg of metformin/kg administered orally twice daily in dogs was well tolerated and equivalent to lower dosage of human (500–2,550 mg/day).^34^ Adverse effects related to metformin were not observed in mice treated with metformin and combination of metformin and X-irradiation. The present study should help inform the selection of dosage of metformin for treatment and prevention of cancer in dogs.

In summary, metformin preferentially radio-sensitized SCs both *in vivo* and *in vitro*, through inhibiting mitochondrial respiration. Additionally, SCs had a higher ability of mitochondrial respiration than ACs, which might account for differences in their radio-sensitivity. Therefore, mitochondrial function might play an important role in the radio-resistance mechanism, and drugs targeting mitochondrial respiration, such as metformin, could be a promising radio-sensitizer in CSCs.

## Materials and methods

### Reagents and treatment

Metformin hydrochloride (metformin), rotenone, CCCP, and oligomycin were obtained from Wako Pure Chemical Industries, Osaka, Japan. The X-irradiation was performed using TITAN-320S (Shimadzu Industrial System, Kyoto, Japan) with a dose rate of 4.17 Gy/min at 200 kVp and 20 mA, using a 2.0-mm aluminum filter for cells, and PANTAK HF-350 (Shimadzu) with a dose rate of 1.30 Gy/min at 200 kVp and 20 mA, using a 0.5-mm aluminum and 0.5-mm copper filter for mice at room temperature.

### Cell culture

The canine osteosarcoma cell line canine osteosarcoma cell line (HMPOS) was used in this study.^35^ ACs were cultured in RPMI (GIBCO by Life Technologies, Grand Island, NY, USA) supplemented with 10% fetal bovine serum (Sigma-Aldrich, St. Louis, MO, USA) and maintained in a humidified atmosphere with 5% CO_2_ at 37°C. Single-cell suspension of ACs was cultured in ultra-low attachment plates (Corning, Corning, NY, USA) in the presence of serum-free DMEM/F12 (Wako) supplemented with 20 ng/mL EGF (Wako) and 10 ng/mL basic FGF (Wako) and maintained in a humidified atmosphere with 5% CO_2_ at 37°C.

### Inhibition of sphere formation

For the evaluation of the effect of metformin on sphere formation, suspension of ACs was seeded at a density of 1 × 10^4^ cells and cultured with 0.1 μM and 0.5 μM metformin for 24 h under the same conditions as those for sphere formation. After 10 days of incubation, the number of spheres >50 μm size were counted.

### Clonogenic cell survival assay

Cells were plated at various concentrations in 60-mm dishes and cultured for 24 h with several concentrations of metformin. Cells were then X-ray irradiated, and the medium was replaced with fresh growth medium. After incubation for 7 days, cells were fixed with methanol and stained with Giemsa’s solution. Each colony consisting of more than 50 cells was scored as a colony-forming unit.

### Intracellular ROS analysis

Intracellular ROS levels were evaluated using a commercial detection kit ROS-ID (Enzo Life Science, Farmingdale, NY, USA). Suspensions of AC and SC were prepared in normal culture flasks and incubated for 24 h, followed by addition of 50 μM metformin 24 h prior to 5 Gy X-irradiation. After irradiation, cells were trypsinized and resuspended in RPMI, and at least 10^5^ cells were analyzed by flow cytometry using FACS Callibur (BD Biosciences, San Jose, CA, USA) according to the manufacturer’s protocol. MFI of each sample was normalized to that of the ACs control. All data were analyzed using the CellQuest software package (BD Biosciences).

### Measurement of OCR by ESR

The ESR was performed as described previously with minor modification.^36^ The peak-to-peak line width of the ESR spectrum of lithium 5, 9, 14, 18, 23, 27, 32, 36-octa-*n*-butoxy-2, 3-naphthalocyanine (LiNc-BuO) shows a liner response to the partial pressure of oxygen (pO_2_) and was used to measure oxygen consumption (Figures S3 and S4). Cells were suspended in 50-μL serum-free RPMI medium containing 0.1 mg LiNc-BuO and 2% dextran. Then, 30 μL of the cell suspension sample was immediately drawn into a glass capillary tube at density 7.5 × 10^4^ per tube. The ESR measurements were carried out using a JEOL-RE X-band spectrometer (JEOL, Tokyo, Japan) with a cylindrical TE011 mode cavity (JEOL).

### Cellar ATP content analysis

Cellular ATP contents were evaluated using the ATP assay kit (Wako) according to the manufacturer’s protocol. The preparation of cells and treatment were performed in the same way as described for ROS evaluation. After irradiation, cells were trypsinized and resuspended in 100-μL serum-free RPMI medium at density of 10^4^ cells per well. Then, 100 μL of ATP assay reagent was added and chemiluminescence from each well was analyzed luminometer (Luminescencer-JNR; ATTO, Tokyo, Japan) set at 25°C.

### Mitochondrial membrane potential analysis

The fluorescent probe TMRM was used for the analysis of mitochondrial membrane potential.^37^ The preparation of cells and treatment were performed in same way as described for ROS evaluation. Cells were incubated with 50 nM TMRM for 30 min at 37°C, trypsinized, and resuspended in RPMI, and at least 10^5^ cells were analyzed by flow cytometry in similar way as described for ROS evaluation.

### Tumor growth inhibition in xenograft mice

7-week-old female BALBcAJc1 nu/nu mice (Hokudo, Sapporo, Japan) were used. Animal experiments and handling were done according to Hokkaido University Institutional Animal Care and Use Committee guidelines (approval number: 18-0086). SCs were prepared at 1 × 10^2^ cells in 50 μL of PBS. Mixture of cells in 50 μL PBS and 50 μL Matrigel (BD Bioscience) were injected subcutaneously into the right hind legs of the mice. The tumor volume (tumor volume = length × [width]^2^ × 0.5) and body weight were measured at the end of the assessment period (tumor length beyond 12 mm). When tumors reached 100 mm^3^ in volume, the mice were randomly assigned to one of the following four groups: (A) control, (B) metformin, (C) X-irradiation, and (D) metformin plus X-irradiation. The group (B) and (D) were treated twice a day with metformin dissoluted in 0.1 mL saline (25 mg/kg twice a day injected intraperitoneally [i.p.]), although the groups (A) and (C) received i.p. injection 0.1 mL of saline twice a day. For the X-irradiation of tumor, the groups (C) and (D) were anesthetized by isoflurane inhalation with oxygen, the body was covered with a 2-mm-thick lead sheet, and tumor-bearing legs were locally exposed to 20-Gy X-irradiation in a single dose after 3 days post-injection of saline or metformin. At the end of the assessment period, mice were humanely sacrificed using inhalation of CO_2_ gas.

### Statistical analysis

For statistical analysis, JMP 14 (SAS Institute, Cary, NC, USA) software was used. All results are represented as the mean ± standard deviation (SD) values. Statistical analysis was performed using the Mann-Whitney’s U test, and differences with p < 0.05 were considered to be statistically significant.

## Supplemental information

Document S1. Supplemental results, supplemental methods, and Figures S1–S4

Document S2. Article plus supplemental information
