# Supplementary material for: Metformin preferentially enhances the radio-sensitivity of cancer stem-like cells with highly mitochondrial respiration ability in HMPOS
Source: Mol Ther Oncolytics. 2021 Aug 19;22:143–51. doi: 10.1016/j.omto.2021.08.007 (PMC8413836; doi:10.1016/j.omto.2021.08.007)
Supplement: Document S1. Supplemental results, supplemental methods, and Figures S1–S4 [file mmc1.pdf]

OMTO, Volume *22*

Supplemental information

Metformin preferentially enhances the radio-sensitivity of cancer stem-like cells with highly mitochondrial respiration ability in HMPOS

Tatsuya Deguchi, Kenji Hosoya, Shango Kim, Yusuke Murase, Kumiko Yamamoto, Tomoki Bo, Hironobu Yasui, Osamu Inanami, and Mahiro Okumura

Supplementary Results

Metformin inhibited DNA Double Strand Break repair of SCs following exposure to X-irradiation

To investigate whether metformin affects the kinetics of DNA double strand breaks (DSB) rejoining, 53BP1 foci were measured after X-irradiation (1 Gy) in SCs and ACs with or without metformin. The peak number of foci was smaller and time-dependent reduction of foci was more rapid in SCs than in ACs. Additionally, metformin increased the peak number of foci and reduced time-dependent reduction of foci (Fig. S1). These results suggested that SCs possess enhanced DNA repair capacity and metformin inhibited the DNA repair especially in SCs.

Metformin did not affect activation of AMPK and its downstream effectors

To evaluate the effect of the AMPK/mTOR signaling pathway, the levels of protein expression and phosphorylation of AMPK, and its downstream targets mTOR, S6K1, and 4EBP1, were measured in ACs and SCs treated with or without metformin (50 μM) 24 hours after X-irradiation (5Gy). In ACs and SCs, levels of AMPK, p-AMPK, and its downstream targets mTOR, p-mTOR, p-S6K1 and p-4EBP1 were comparable with or without metformin, X-irradiation or metformin plus X-irradiation (Fig. S2).

Supplementary Methods

Analysis of DNA kinetics by Immunofluorescence staining for p53-binding protein 1

The immunofluorescence staining for p53BP1 was performed as described previously with minor modification.[1] Suspension of AC and SC were seeded in a slide and cultured for 24 hours with or without 50 μM metformin. At the indicated time after X-irradiation (1Gy), cells were fixed with 4% paraformaldehyde (Wako) for 20 minutes at RT. Cells were permeabilized with PBS containing 0.5% Triton X-100 for 5 minutes 4°C and blocked with PBS containing 6% goat serum for 30 minutes at RT. The blocked cells were incubated with a rabbit anti- p53BP1 antibody (Abcam) at 1:2,000 dilution in 3% goat serum overnight at 4°C and then incubated in the dark with an Alexa Fluor 488-conjugated anti-rabbit secondary antibody (Abcam) at a 1:2,000 dilution for 90 minutes. After incubation, they were counterstained with Prolong® Diamond Antifade Mountant with 4', 6'-diamidino-2-phenylindole (DAPI) (Thermo Fisher Scientific). Fluorescence microscopic analysis was performed using an Zeiss LSM 700 confocal laser microscope (Zeiss) with reflected light fluorescence, and foci were counted using Image J software (National Institutes of Health).

Analysis of signaling pathway by Western blotting

The preparation of cells and treatment were performed in same way as described for ROS evaluation. Cells were collected and lysed with 1×SDS sample buffer (2% SDS, 10% glycerol, 6% β-mercaptoethanol, 50 mM Tris pH 6.8, and 0.001% bromophenol blue). Then sample were loaded on 5 and 10% w/v SDS-polyacrylamide gel, and transferred to the Whatman Protran nitrocellulose membrane (Merck) and the ImmobilonTM-P Transfer Membrane (Merck). Nonspecific antibody binding was blocked with 3% milk in PBS with Tween 20 for 30 minutes at RT with shaking. Membranes were incubated in primary antibody overnight on a shaker at 4˚C. Membranes were washed with a PBST buffer then incubated in HRP-conjugated secondary antibody (Thermo Fisher Scientific, dilution 1:10,000 in PBST with 1% milk) for 1 hour on a shaker at RT. Membranes were washed as before and then incubated in HRP substrate for 5 minutes at RT. The protein antibody reaction were visualized with Western BLoT Ultra Sensitive HRP Substrate (TaKaRa Bio) and detected using an ImageQuant LAS-4000 mini system (GE Healthcare Japan). The following primary antibodies were used: polyclonal rabbit anti-phospho-AMPK α1 antibody (Thr172, #07-626) (Millipore, MD, USA), anti-AMPK α1 antibody (#07-350), anti-mTOR antibody (#2983) (Cell Signaling Technology Japan), anti-phospho-mTOR

antibody (Ser2448, #5536), TSC2 antibody (#4308), anti- phospho-4EBP1antibody (Thy37/46, #2855), anti-phospho-70S6K antibody (Thr389, #9234), and β-actin (#4967). These antibodies were diluted 1:5,000 (TSC2) or 1:3,000 (the other antibodies) in PBST with 1% milk. These cross-reaction of these antibodies with canine molecules have been reported.[2]

Measurement of oxygen consumption ratio by electron spin resonance (ESR)

The ESR was performed as described previously with minor modification.[3] In ESR spectra each line-width were measured as Fig. S3. The spectral line width was analyzed using a Win-Rad radical analyzer system (Radical Research, Tokyo, Japan). Lithium 5, 9, 14, 18, 23, 27, 32, 36-octa-*n*-butoxy-2, 3-naphthalocyanine (LiNc-BuO) were suspended in PBS equilibrated with mixtures of oxygen/nitrogen gases. The ESR line width versus oxygen partial pressure (pO_2) calibration curve was shown in Fig. S4.

Reference

[1] Deguchi, T, Hosoya, K, Murase, Y, Koangyong, S, Kim, S, Okumura, M. (2019). Analysis of radiosensitivity of cancer stem-like cells derived from canine cancer cell lines, *Vet. Comp. Oncol.*, 17, 119-129.

[2] Saeki, K, Watanabe, M, Tsuboi, M, Sugano, S, Yoshitake, R, Tanaka, Y, Ong, S, M, Saito, T, Matsumoto, K, Fujita, F, et al. (2015). Anti-tumour effect of metformin in canine mammary gland tumour eclls, *Vet. J.*, 205, 297-304.

[3] Yamamoto, K, Yasui, H, Bo, T, Yamamori, T, Hiraoka, W, Yamasaki, T, Yamada, K, Inanami, O. (2018). Genotoxic Responses of Mitochondrial Oxygen Consumption Rate and Mitochondrial Semiquinone Radicals in Tumor Cells, *Appl. Magn. Reson.*, 49, 837-851.

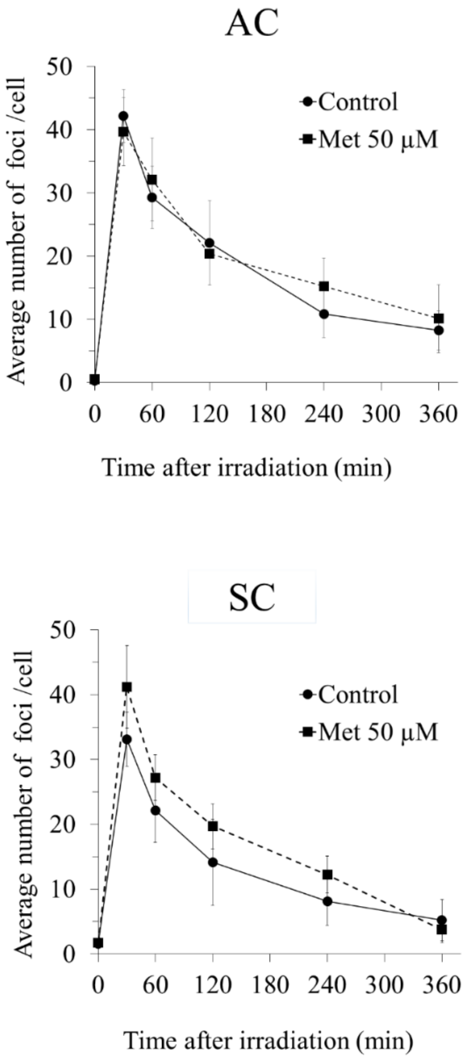

Fig.S1. DNA repair kinetics after X-irradiation with or without metformin in ACs and SCs

Analysis of DNA repair kinetics for the formation of 53BP1 foci treated with or without metformin in ACs and SCs after 1 Gy X-irradiation. These results were analyzed using Mann-Whitney *U* test. $*p < 0.05$ for control versus metformin.

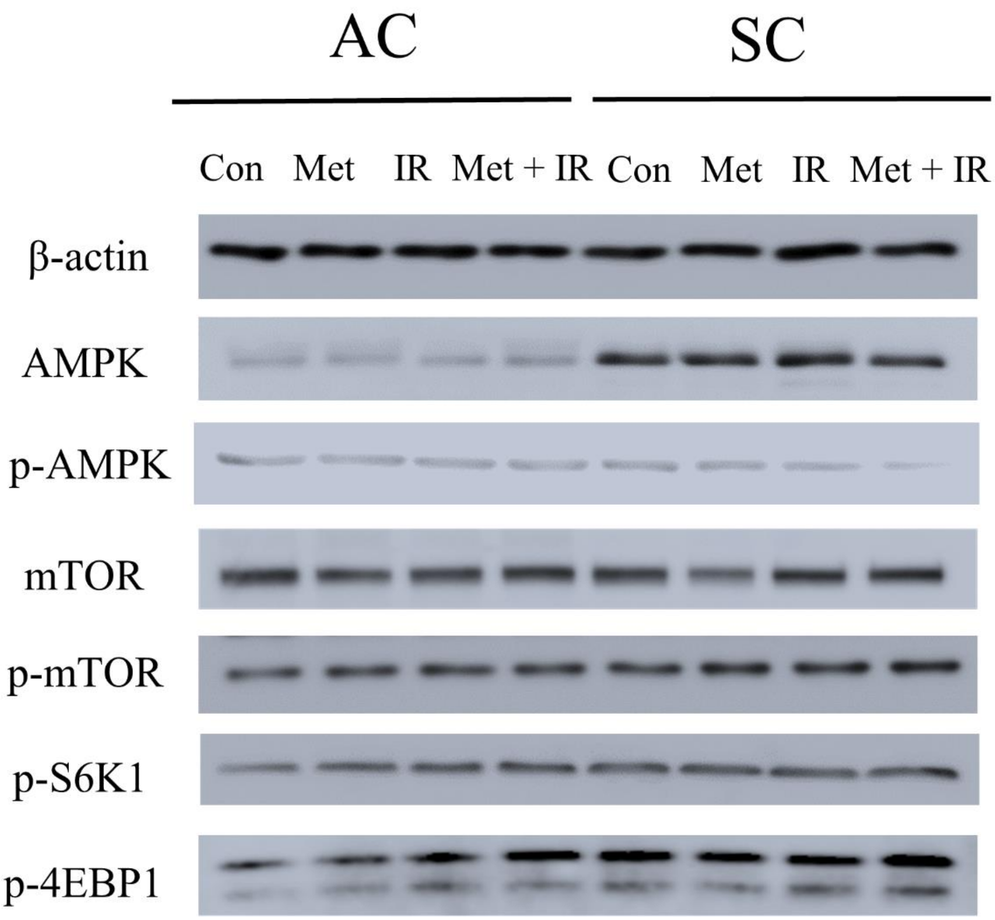

Fig. S2. Effect of metformin on the phosphorylation of the AMPK/mTOR signaling pathway

ACs and SCs were after 24 hours exposure with 50 μM metformin (Met) or 5 Gy X-irradiation (IR) or both metformin plus X-irradiation (Met + IR).

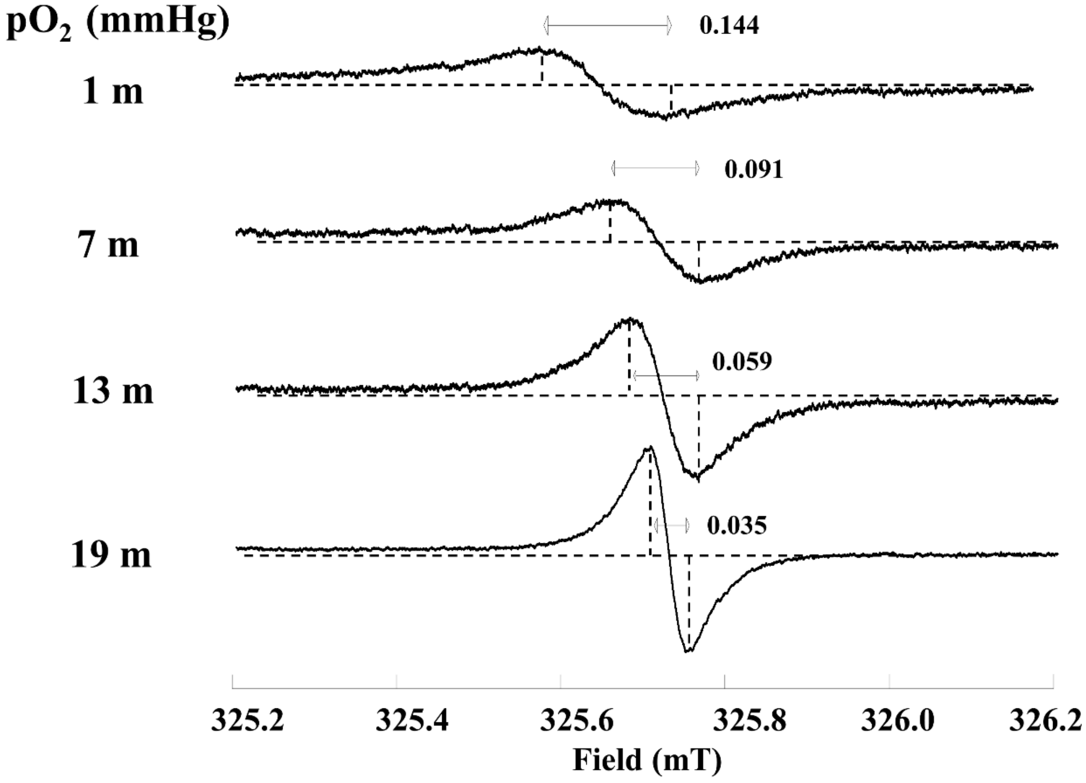

Fig. S3. Measurement of peak-to-peak line width of ESR spectra

Representative ESR spectra obtained from the medium containing LiNc-BuO (2 mg/mL), ACs of HMPOS cells after X-irradiation, and 5% dextran.

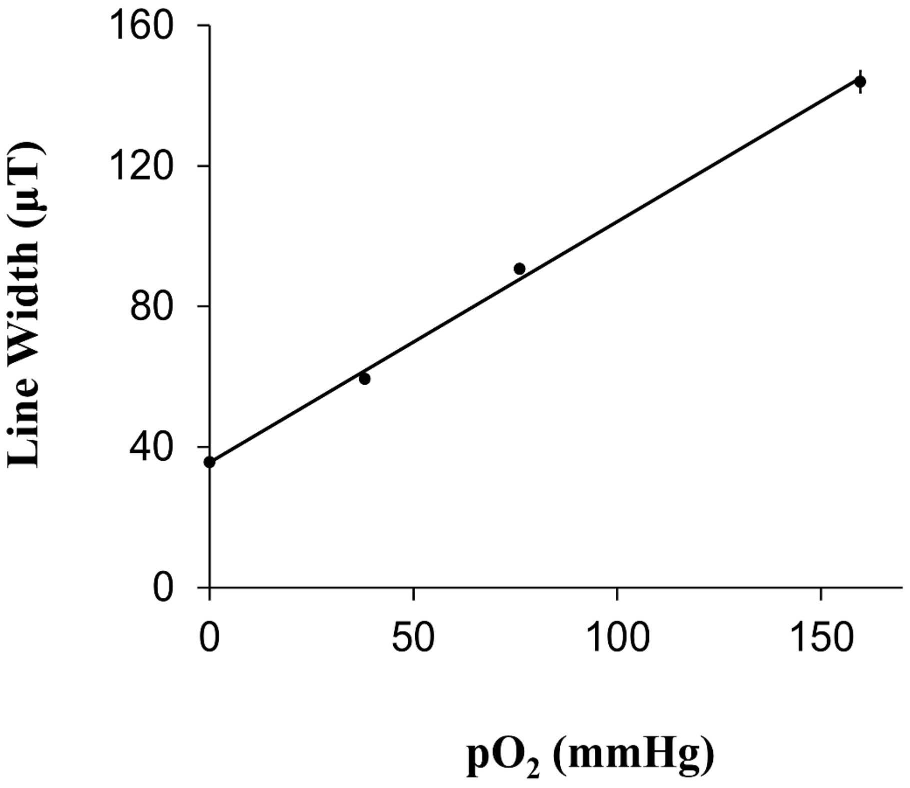

Fig. S4. Calibration curve of pO2 in the medium as to line width (µT) of ESR spectrum

Each dot and vertical bar indicates mean±standard error. The approximation formula, line width (mT)=0.685×pO2 (mmHg)+35.558, was obtained using least-squares function approximation and the coefcient of determination (R^2) was 0.9976.
